# Association Between Smoking and Premenstrual Syndrome: A Meta-Analysis

**DOI:** 10.3389/fpsyt.2020.575526

**Published:** 2020-11-26

**Authors:** So Hee Choi, Ajna Hamidovic

**Affiliations:** College of Pharmacy, University of Illinois at Chicago, Chicago, IL, United States

**Keywords:** smoking, premenstrual syndrome, premenstrual dyspohoric disorder, luteal, affect

## Abstract

Results of basic science studies demonstrate shared actions of endogenous neuroactive steroid hormones and drugs of abuse on neurotransmission. As such, premenstrual syndrome (PMS) may be associated with smoking, however, results from studies examining this relationship have been mixed. Following PRISMA guidelines, we extracted unique studies examining the relationship between smoking and PMS. We used the escalc () function in R to compute the log odds ratios and corresponding sampling variance for each study. We based quality assessment on the nature of PMS diagnosis and smoking estimation, confounding adjustment, participation rate, and *a priori* specification of target population. Our final sample included 13 studies, involving 25,828 study participants. Smoking was associated with an increased risk for PMS [OR = 1.56 (95% CI: 1.25–1.93), *p* < 0.0001]. Stratified by diagnosis, the effect size estimate was higher for Premenstrual Dysphoric Disorder (PMDD) [OR = 3.15 (95% CI: 2.20–4.52), *p* < 0.0001] than for PMS [OR = 1.27 (95% CI: 1.16–1.39), *p* < 0.0001]. We review some of the basic mechanisms for the observed association between smoking and PMS. Given nicotine's rewarding effects, increased smoking behavior may be a mechanism to alleviate affective symptoms of PMS. However, smoking may lead to worsening of PMS symptoms because nicotine has effects on neurocircuitry that increases susceptibility to environmental stressors. Indeed, prior evidence shows that the hypothalamic-pituitary-adrenal (HPA) axis is already sub-optimal in PMS, hence, smoking likely further deteriorates it. Combined, this complicates the clinical course for the treatment of both PMS and Tobacco Use Disorder in this population.

## Introduction

The rhythmicity of sex hormones, which drives the menstrual cycle, signifies an essential life physiological pattern. Its synchronization with external environment and internal stimuli promotes dynamic stability; however, its perturbations are associated with disorderly states, such as premenstrual mood disturbance (1, 2).

The luteal phase of the menstrual cycle is comprised of rising estrogen and peaking progesterone, with the levels of both sex hormones falling toward the end of this phase. Multiple changes on the molecular, system, clinical and behavioral levels take place, and, in fact, the luteal phase may be considered a “normally stressed physiology,” though individual differences are pronounced and amplified (3). Changing sex hormones in the luteal phase are associated with decreased levels of amino acids and lipid species, possibly indicative of an anabolic state (4). During this time women may experience worsening of affect, insomnia, diabetes, and inflammatory bowel disease (5, 6).

Interestingly, women who smoke also tend to increase their nicotine intake during the luteal phase of the menstrual cycle, though the results have not been consistent. Whereas DeBon et al. (7) and Sakai and Ohashi (8) observed an increase in the number of cigarettes smoked in the luteal phase compared to the ovulatory phase or the follicular phase, respectively, Allen et al. (9) reported no such increase. The differences in findings to date may be related to lack of ovulation confirmation (suggesting insufficient bounds of follicular and luteal phases), absence of objective nicotine intake measures, inclusion of women with underlying health conditions and age-related differences (10).

In some instances, sex hormones have opposite effects on smoking behavior. For example, estrogen has been shown to increase rewarding value of nicotine (11), while progesterone diminishes motivation for nicotine (12). There is a strong relationship between high estrogen levels, observed in premenopausal women, and increased metabolism of nicotine (13, 14). As increased nicotine metabolism is associated with poor smoking cessation outcomes (15) and higher smoking rates (16), estradiol's metabolism-promoting effect on nicotine may be one factor underlying the lower smoking cessation rates in women (17), though this hypothesis is yet to be formally tested. Furthermore, animal models show that menstrual cycle hormonal changes have significant impact on nicotine withdrawal, with estradiol promoting and progesterone reducing anxiety-like behaviors resulting from nicotine withdrawal (18).

In addition to being associated with a myriad of symptoms and worsening of several disease states, the late luteal phase is associated with the Premenstrual Syndrome. PMS is one of the most common health problems reported in women of reproductive age, and estimated to affect 20–30% of this population (19). It refers to a cluster of adverse affective and physical symptoms experienced in the late luteal phase which resolve by the end of menstruation (20). The most common affective symptoms include irritability, anxiety, sadness/depression and hopelessness, while the most common physical symptoms include swelling/bloating, breast tenderness, aches/joint pains, and cramps (21). The syndrome is associated with disability, work impairment, disrupted activities and personal relationships over many years of menstrual cycling (22). PMS diagnosis, according to the *Diagnostic and Statistical Manual of Mental Disorders 5* (*DSM-5*), is made when, between 1 and 4 affective, behavioral or physical symptoms are experienced specifically in the premenstruum. PMDD is considered the more severe form of PMS, and the diagnosis requires that severity of a combination of 5 (out of total 11) physical, psychological and behavioral symptoms increases in the premenstrual period. At least one of these symptoms is required to be affective in nature. Our understanding of PMS etiology is limited, despite the conditions' long historical presence.

Sex hormones have been implicated in the pathophysiology of PMS due to a significant temporal relationship between symptoms and onset of menstruation. Indeed, women with and without PMS have similar reproductive hormone [i.e., estradiol, progesterone, luteinizing hormone (LH), and follicle stimulating hormone (FSH)] levels, when measured cross-sectionally. Similarly, no consistent diagnosis-related differences can be found for progesterone-derived neurosteroids allopregnanolone (ALLO) and pregnanolone (23, 24). Despite normal hormone levels in women with PMS, the possibility that dynamic hormonal events (i.e., changing ovarian hormone levels) during the menstrual cycle are implicated in the etiology of PMS has been suggested from results of prospective cohort studies (25–27).

Reproductive hormones influence neurotransmission in the dopaminergic system (28). For example, animal research investigating sex differences in drug reward provides compelling evidence that estrogen administration increases, and ovariectomy dampens, operant behavior in female animals (29, 30). However, though etiology of PMS is thought to be related to the altered trajectories of neuroactive steroids, what those steroids are, and if they include estradiol in particular, is not known at this time. Therefore, although it is plausible—based on mechanistic literature—to speculate that there may be a relationship between drug intake (i.e., smoking) and premenstrual mood disturbance, this relationship has not been established on the epidemiologic level.

A number of studies examining lifestyle factors which influence risk for PMS have reported their findings, and have shown no relationship (31, 32) as well as statistically significant association (33, 34) between smoking and PMS. The purpose of this meta-analysis is to pool the results of studies to date, in order to determine the effect size of the relationship, should it exist on a statistically significant level.

## Materials and Methods

### Search Strategy

We conducted a literature search in PubMed, PsycINFO, and Web of Science for eligible studies published through April 3, 2020. The two authors completed their search independently according to the Preferred Reporting Items for Systematic Reviews and Meta-Analyses (PRISMA) guidelines (35). They reconciled any discrepancies by reviewing the literature jointly for specific points of difference. For PubMed and PsycINFO, we searched in the following fields: Premenstrual (All Fields) AND {[“smoking”(All Fields)] OR [“nicotine”(All Fields)]}. For Web of Science, we searched topic of [Premenstrual AND (smoking or nicotine)] in all years and following indexes: SCI-EXPANDED, SSCI, A&HCI, ESCI. We searched the references of all final papers for additional sources of data.

### Study Selection

This meta-analysis was conducted in order to evaluate the association between current smoking (Y/N) and current PMS (Y/N). We included original data abstracts from cohort, case-control or cross-sectional studies. Study abstracts were excluded if they: (1) did not specify PMS or PMDD as an outcome of interest, (2) did not specify smoking, nicotine or tobacco as an exposure factor, (3) described results of non-human research studies, (4) were review papers, letter, comments, or books, (5) described results of a laboratory study involving nicotine administration or a smoking cessation trial, and (6) repeated the same study samples across multiple publications. In the event the same sample was reported across several publications, we selected the most applicable study.

### Quality Assessment

In order to assess quality across studies, we developed a 5-point scale form Fernandez et al. (36) to synthesize the following information:

1. PMS diagnosis. If the diagnosis was ascertained through standard or validated questionnaire, or if a prospective measurement was implemented, a study was coded as 1. If the questionnaire was non-standardized, or not mentioned, a study was coded as 0.
2. Measurement of smoking cigarettes. If smoking was assessed through standard or validated questionnaire, or if a prospective measure was implemented, a paper was coded as 1. Otherwise, if it was a non-validated questionnaire, or there was no information regarding ascertainment of this information, a study was coded as 0.
3. Confounding assessment. If the effect estimator was adjusted for age, at a minimum, a paper was coded as 1. Otherwise, we coded a study as 0.
4. Participation. If participation (i.e., retention) exceeded 80% of potential participants, a paper was coded as 1. Otherwise, it was coded as 0.
5. Target population: If the target population was clearly defined, a paper was coded as 1. Otherwise, if participation was based on convenience sampling of subjects, such as patients of a single consultation or volunteers, or if participation was not explained, a paper was coded as a 0.

### Data Analysis

The goal of the present meta-analysis was to aggregate the results from selected studies contrasting two groups (smoker vs. non-smoker), with each study measuring a dichotomous outcome of interest (PMS vs. non-PMS). The effect size measure used to quantify the size of the group difference was the odds ratio. For each study selected for the present meta-analysis, we extracted the estimate of the effect measure that was adjusted for the largest number of confounders, recording what those confounders are (Table 1). We weighted the study-specific log odds ratios by the inverse of their variance to compute a pooled estimate.

**Table 1:** Study Information.

**First author**	**Year**	**Country**	**Study design**	**Sample size**	**Case/Control**	**Sample characteristics**	**PMS/PMDD**	**Prevalence**	**PMS assessment method**	**Adjustment, restriction or matching factors**	**Smoking assessment method**
Acikgoz	2017	Turkey	Cross-sectional	618	359/259	University students	PMS	58.1%.	PMSS (R)	ND	Self-report (Smoking: Y/N)
Alpaslan	2014	Turkey	Cross-sectional	308	205/103	University students	PMS	66.6%	PAF (R)	Adjustment (age, BMI, smoking and state of regular exercise)	Self-report (Smoking: Y/N)
Bryant	2006	United Kingdom	Case-control	58	31/27	General population	PMS	_	DRSP (P-1 Cycle)	Adjustment (age and BMI)	Self-report (Current/Former/Never)
Chuong	1995	United States	Case-control	372	190/182	Patients	PMS	_	Medical history and prospective symptom charting (P-1 Cycle)	ND	Self-report (Smoking: Y/N and quantity for Y)
Cohen	2002	United States	Cross-sectional	513	33/480	General population	PMDD	2.7%	DRSP (P-3 Years)	Adjustment (age, race, age at menarche, and a past history of depression)	Self-report (Current/Former/Never)
Deuster	1999	United States	Cross-sectional	874	70/849	General population	PMS	8.2%	SPAF (R)	Adjustment (race, age, age at menarche, length of menses, BMI, education, intake of alcoholic beverages, pack-years of smoking, stress score, nutrition, and physical activity)	Self-report (Smoking: Y/N and quantity for Y)
Forrester-Knauss	2011	Switzerland	Cross-sectional	3,518	413/2,848	General population	PMS	11.7%	PSST (R)	ND	Self-report (Current/Former/Never)
Hong	2012	South Korea	Cross-sectional	2,499	59/2,440	General population	PMDD	2.4%	WHO-CIDI (R)	Adjustment (age)	CIDI Interview (Nicotine Dependence Y/N)
Pilver	2011	United States	Cross-sectional	2,590	110/2,480	General population	PMDD	4.4%	WMH-CID (R)	Adjustment (race)	Self-report (Current/Former/Never)
Pinar	2011	Turkey	Cross-sectional	316	228/88	University students	PMS	72.1%	PMSS (R)	ND	Self-report (Smoking: Y/N)
Sadler	2010	United Kingdom	Cross-sectional	974	234/740	General population	PMS	24.0%	Menstrual symptom diary (P-1 cycle)	Adjustment (age, BMI, education, smoking, tress, and contraceptive use)	Self-report (Smoking: Y/N)
Skryzpule-Plinta	2010	Poland	Cross-sectional	1,540	32/1,508	General population	PMDD	2.0%	Menstrual symptom diary (P-2 cycles)	ND	Self-report (Smoking: Y/N and duration of smoking and number of cigarettes information)
Strine	2005	United States	Cross-sectional	11,648	2,213/9,435	General population	PMS	19.0%	Self-report (R)	Adjustment (age, race or ethnicity, education, marital status, and employment status)	Self-report Non-smoker vs. Current smoker: having smoked >100 cigarettes during their lifetime and who currently smoked every day or some days.

*ND, Not discussed; PMS, Premenstrual Syndrome; PMSS, Premenstrual Syndrome Scale; PAF, Premenstrual Assessment Form; DRSP, Daily Symptoms Report; BMI, Body Mass Index; SPAF, Shortened Premenstrual Assessment Form; PSST, Premenstrual Symptoms Screening Tool; WHO-CIDI, World Health Organization Composite International Diagnostic Interview; WMH-CIDI, World Mental Health Composite International Diagnostic Interview; NHIS, National Health Interview Survey; R, Retrospective assessment; P, Prospective Assessment; CIDI, Composite International Diagnostic Interview*.

When data was presented in a 2 × 2 format, we used the escalc () function of the Metafor package in R to compute the log odds ratios (and corresponding sampling variance) for each study. The escalc () function directly computes the log-transformed odds ratios, as these are the values we needed for the meta-analysis. For studies reporting odds ratio with *p*-values directly, we transformed the values to log odds, converted the *p*-values into corresponding z scores, confidence interval bounds and standard errors. In the final step, we fit a random-effects model to these data with the rma () function of the Metafor package in R.

We assessed source-study heterogeneity using the χ^2^-based *Q*-test with its associated *p*-value. A statistically significant Q statistic suggests different effect sizes across studies, implying that methodological or population sample differences may be introducing variance across individual studies. We quantified heterogeneity using *I*^2^ with values 25, 50, and 75% suggestive of small, medium and large heterogeneity and calculated potential publication bias using the ranktest () function (rank correlation test for funnel plot asymmetry). We completed sub-analyses according to validated region of study (US/Western Europe or Other), PMS/PMDD diagnosis, quality score (<3 or ≥3), and mean age. We attempted to extract as much additional information as possible about additional sources of heterogeneity in the moderator analysis. However, the number of studies cross listing the same variables was limited in insufficient for moderator analysis. For example, body mass index (BMI) was available in only four studies, duration of menstruation and age of menarche in only three studies, and psychiatric co-morbidity in three studies. Nonetheless, our moderator analysis had sufficient data to conduct four moderator analyses. Finally, we conducted further analyses on the three moderators (geographical region, quality, and mean age) according to the condition (PMS vs. PMDD).

## Results

### Characteristics of Individual Studies

**Figure 1:**
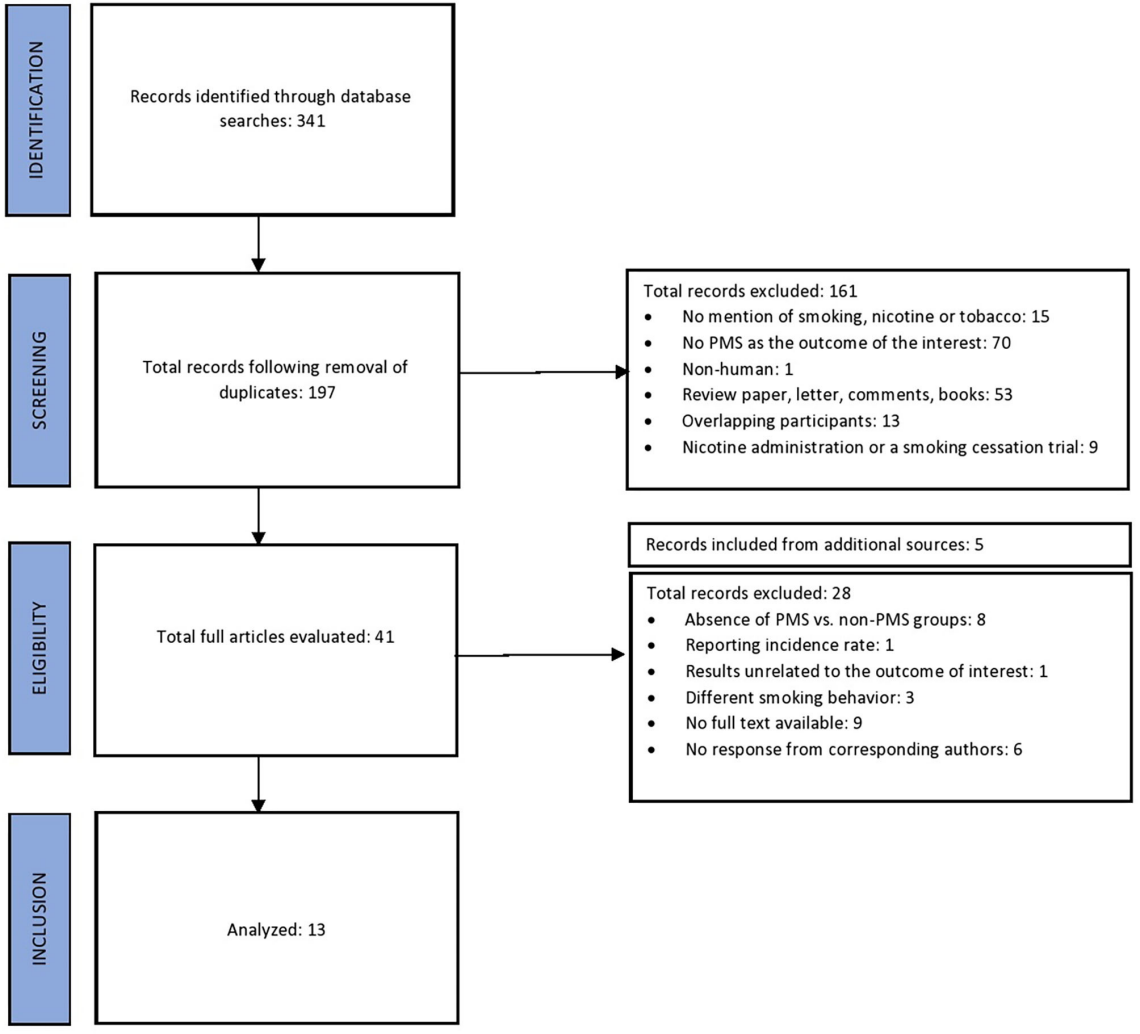
PRISMA flow diagram.

Following removal of duplicates, we identified 197 manuscripts, with the total of 13 final studies contributing data for the present meta-analysis. Results of our search are displayed in PRISMA Figure 1.

As shown in Table 1, eight studies were conducted in the United States/Western Europe, three in Turkey and one in South Korea. Two studies were case-control and the rest were cross-sectional. The combined sample size, reflecting university students, general population and patients, was 25, 828 participants. Four studies evaluated PMDD and nine studies evaluated PMS. Five studies reported no adjustment for the effect estimator.

### Evaluation of Pooled Log Odds Ratio

**Figure 2:**
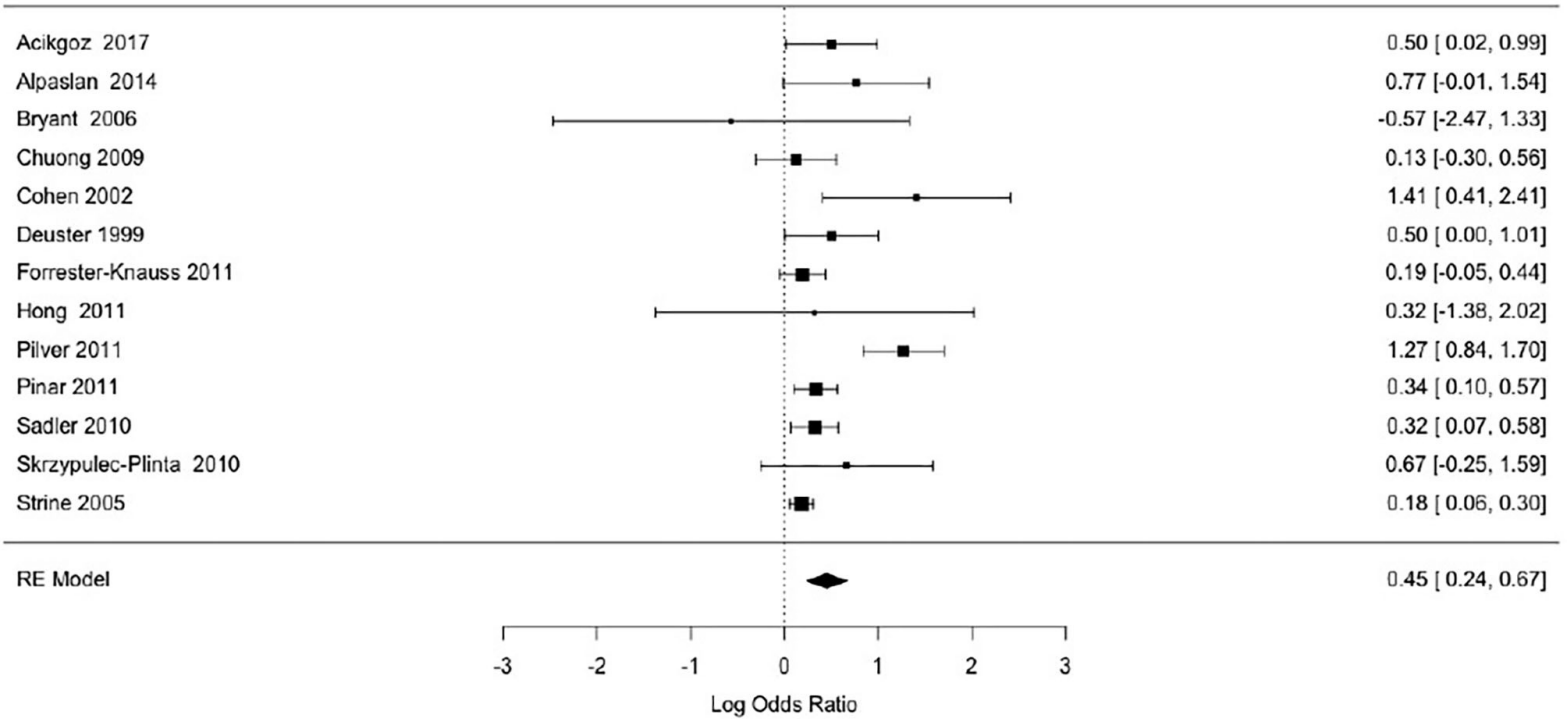
Forest plot of association between smoking and PMS for the random effects (RE) model.

**Figure 3:**
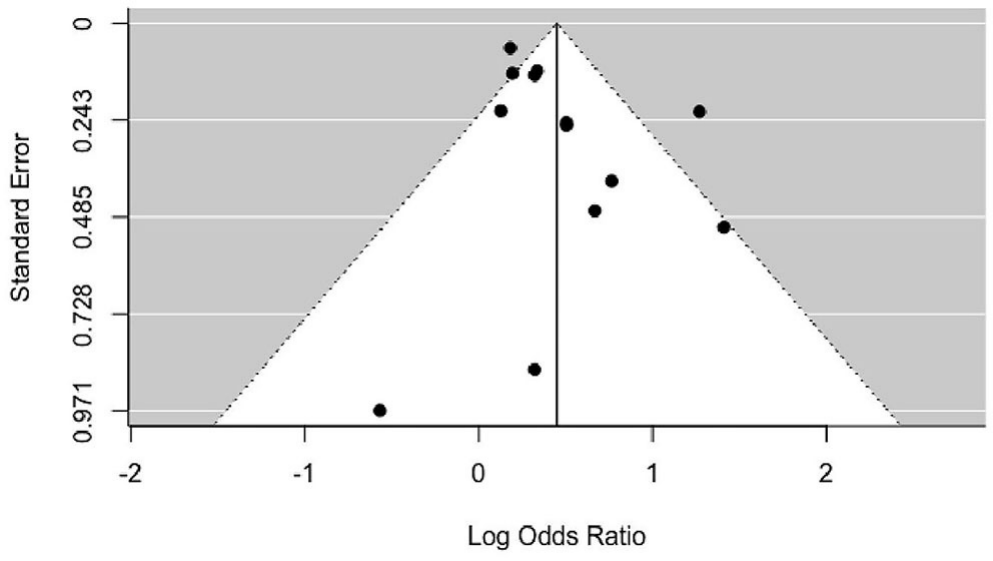
Funnel plot of included studies. Results of statistical analysis indicate absence of asymmetry and publication bias (*p* = 0.43).

Smoking was associated with a moderate increase of the risk for PMS (OR = 1.56, *p* < 0.0001) for the random effects model (Figure 2). The confidence interval range was between 1.25 and 1.93. Significant between-study difference was detected (tau= 0.28; *H*^2^ = 3.74), with the level of heterogeneity in the medium-high range (*I*^2^ = 73.28%, *Q* = 33.15; *p* < 0.001). The Rank Correlation Test for Funnel Plot Asymmetry resulted in Kendall's tau of 0.17 (*p* = 0.43), indicating absence of small study effects (Figure 3).

### Source of Heterogeneity Examination

Addition of diagnosis (PMDD vs. PMS) substantially decreased heterogeneity to *I*^2^ = 1.85%, QE = 9.5; *p* = 0.57 (Table 2). Residual heterogeneity remained statistically significant when the model was evaluated by region and quality score. Stratified by diagnosis, PMDD showed a stronger association with smoking than PMS (PMDD [OR=3.15 (95% CI: 2.20–4.52), *p* < 0.0001] than for PMS [OR = 1.27 (95% CI: 1.16–1.39), *p* < 0.0001]. Sub-analysis of studies stratified by quality showed that lower quality studies did not show a significant association between PMS and smoking (Table 2). Mean age was not a statistically significant moderator (Supplementary Material). Residual heterogeneity was reduced to 0% in the moderator analysis for region, for both PMS and PMDD (Supplementary Table 2).

**Table 2:** Results of heterogeneity and moderator analyses.

**Study characteristics**		**Number of studies**	**OR (95% CI), *p*-value**	**Test for heterogeneity**	***I*^**2**^ residual heterogeneity**	**Test for residual heterogeneity**	**Test of moderators**
Region	United States/Western Europe	8	1.56 (1.12–2.18), *p* < 0.001	*Q* = 22.80, *p* < 0.001	74.00%	QE = 31.62, *p* < 0.001	QM = 15.54, *p* < 0.001
	Other	5	1.49 (1.22–1.82), *p* < 0.0001	*Q* = 1.64, *p* = 0.80			
Disorder	PMS	9	1.27 (1.16–1.39), *p* < 0.0001	*Q* = 6.98, *p* = 0.53	1.85%	QE = 9.50, *p* = 0.57	QM = 69.01, *p* < 0.0001
	PMDD	4	3.15 (2.20–4.52), *p* < 0.0001	*Q* = 2.52, *p* = 0.47			
Quality	<3	4	1.71 (0.99–3.00), ns	*Q* = 20.01, *p* < 0.001	73.95%	QE = 31.56, *p* < 0.001	QM = 15.76, *p* < 0.001
	≥3	9	1.39 (1.19–1.59), *p* < 0.0001	Q = 11.54, *p* = 0.17			

## Discussion

The present meta-analysis pools results of literature to date to examine whether association between smoking and PMS exists, and, if so, to establish its effect size. We demonstrate a statistically significant association between smoking and PMS. The overall effect size is in the medium range, however, as expected, when stratified by PMS vs. PMDD, the effect size for PMDD was larger. In addition, stratified by quality score, studies with low quality scores no longer showed statistical significance between smoking and PMS.

There may be several reasons for the observed association between smoking and PMS. Premenstrual syndrome, like Major Depressive Disorder, is classified in the Mood Disorders section in DSM-5. The most commonly reported affective symptoms by women suffering from PMS include irritability, anxiety, sadness/depression, and hopelessness. Indeed, depressed smokers commonly report that smoking regulates their negative mood states (37, 38). Evaluating the relationship between depressive symptoms and nicotine dependence in a sample of 202 participants, Lerman et al. (39) demonstrated that negative affect reduction and stimulation are mediators of this relationship. Nicotine's rewarding effects are mediated via serotonergic, cholinergic and dopaminergic systems. It activates the mesolimbic dopamine system through its actions at the nicotinic receptors of the ventral tegmental area neurons (40, 41). In addition, nicotine reinforcement is modulated by the 5-HT neurotransmission, as 5-HT3 receptor antagonists block nicotine place preference (42). Therefore, dysphoria resulting from symptoms induced by PMS, may lead to increased smoking behaviors as a mechanism to alleviate it.

However, smoking may lead to development of PMS, or worsen affective symptoms in women with PMS, because nicotine has effects on neurocircuitry that increases susceptibility to environmental stressors. Acutely, nicotine potentiates the hypothalamic-pituitary-adrenal (HPA) axis, resulting in hypersecretion of cortisol and alterations in the activity of the associated monoamine neurotransmitter system (43–45). However, repeated administration of nicotine results in neuroadaptations which eventually oppose the acute effects of drugs (46–48). Smokers have a blunted HPA stress response (49) and the attenuated HPA response to stress predicts shorter time to relapse (50). A finding that that chronic smoking is associated with dysregulation of the HPA stress response is further complicated in PMS patients, who also have a blunted response to stress. In a human experimental study contrasting acute stress reactivity between women with PMS and healthy controls, Huang et al. (51) demonstrated a blunted cortisol output in women diagnosed with PMS. Interestingly, the cortisol output, rather than heart rate or subjective response to stress, significantly correlated with symptoms of PMS, suggesting that hypo-reactivity of the HPA axis predicts PMS severity. Combined with findings of the hypo-reactive HPA axis shown in smokers, prolonged nicotine intake further deteriorates functionality of stress response in PMS, thereby complicating the clinical course of both Tobacco Use Disorder and PMS in this population.

Our analysis uncovered that studies with small sample sizes generally reported non-significant relationship between smoking and PMS (Figure 2). For example, the number of cases studies by Skrzypulec-Plinta et al. (32), Bryant et al. (52) and Hong et al. (53) was below 60, raising the possibility that the studies were underpowered to detect a significant association. Larger studies, including at least 100 cases, tended to show a statistically significant relationship between smoking and PMS. Our meta-analysis provides meaningful information for future studies needing accurate estimates in order to carry out power calculations.

Interestingly, prevalence of PMDD in the current sample was consistent across studies, but varied greatly for PMS. For PMDD, prevalence ranged from 2.0 to 4.4%, and was based on either the World Mental Health Composite International Diagnostic Interview (WMH-CIDI) (33, 53) or a prospective diary rating of symptoms (32, 54). While the prospective rating is the gold standard for determination of either PMDD or PMS, the WMH-CIDI is consistent with DSM-IV diagnostic criteria and has been successfully applied in multiple studies (55–58). Our meta-analysis shows that application of either methodology yields a similar estimate for PMDD. The estimation of PMS prevalence, however, is more complicated, and in our study ranged from 8.2 to 72.1%. The study which detected a prevalence rate of 8.2% (59) utilized a modified version of the Premenstrual Symptom Screening Tool (60), which was originally developed to detect PMDD. Therefore, the study recruited participants with moderate to severe form of PMS. The study reporting PMS prevalence of 72.1% (29) utilized the Premenstrual Syndrome Scale (PMSS) (61) in which participants rate physical, psychological, and behavioral symptoms on a 5-point Likert scale. The range is between 44 and 220, and participants had to score > 110 to be classified as having PMS. Therefore, a number of women with mild PMS, along with women with more severe form of PMS were recruited, increasing the prevalence found in this study. In our opinion, a prospective measure of PMS symptoms, detecting an increase in the premenstruum, and resolution by the end of menstruation yields the most accurate estimate of 24%, as was found in the study by Sadler et al. (62). This prevalence (20–30%) is also generally accepted in the field (19).

The current analysis presents limitations. As the prevalence of PMS ranged considerably across studies, it is possible that the odds ratio estimate (OR = 1.56) and the confidence interval (1.25–1.93) may not be precise. However, a finding (36) of an association between PMS and alcohol consumption showing an odds ratio of 1.45 (CI: 1.17–1.79) suggests that, at a minimum, the statistically significant association found in the present meta-analysis is valid. The effect size for PMDD is likely more precise than the effect size for PMS, based on the concise estimate of PMDD prevalence found across different studies. A general limitation of the meta-analytic approach is lack of complete data availability. The availability of data from publications in our analysis was 30%. Despite this challenge, in our sample involving 25,828 study participants, we found no evidence of publication bias. We detected a medium-high range between-study heterogeneity in the main analysis. We have identified some of the sources of that heterogeneity. As would be expected, heterogeneity was non-significant in our analyses evaluating association with smoking for PMS and PMDD separately. Moreover, stratified by our quality assessment score, heterogeneity was non-significant in the studies (*n* = 9) assessed to be of high quality. This detection points to the separate clinical constructs for PMS vs. PMDD, as well as rigor of the quality assessment tool we implemented in the present study. In addition, our analysis draws attention to the methodology of assessing smoking in the present literature. Only three studies included in the meta-analysis described their smoking assessment methodology, with the remaining studies only assessing smoking as binary measure (current smoking: Y/N). The study by Skryzpulec-Plinta et al. (32), for example, thoroughly assessed smoking by both binary and continuous measures. The investigators assessed the duration of smoking as well as the number of cigarettes per day. Having this information from all the studies would have refined our analysis, demonstrating a hypothesized dose-response relationship between PMS and smoking.

In the present meta-analysis, we found that across studies smoking is associated with an increase of the risk of PMS. This finding addresses a gap in the literature related to smoking as an addictive behavior in women exhibiting premenstrual disturbance, and contributes to a greater understanding of the clinical course in this population.

## Data Availability Statement

The original contributions presented in the study are included in the article/Supplementary Materials, further inquiries can be directed to the corresponding author/s.

## Author Contributions

AH and SC completed a literature search, data gathering, and statistical analysis and wrote the manuscript. Both authors contributed to the article and approved the submitted version.

## Conflict of Interest

The authors declare that the research was conducted in the absence of any commercial or financial relationships that could be construed as a potential conflict of interest.
