# Supplementary material for: Association Between Smoking and Premenstrual Syndrome: A Meta-Analysis
Source: Front Psychiatry. 2020 Nov 26;11:575526. doi: 10.3389/fpsyt.2020.575526 (PMC7725748; doi:10.3389/fpsyt.2020.575526)
Supplement: Supplementary file 2 [file Data_Sheet_2.docx]

Supplementary Material:

**Result of Moderator Analysis for Mean Age**

Mixed-Effects Model (k = 9; tau^2 estimator: REML)

tau^2 (estimated amount of residual heterogeneity): 0.1159 (SE = 0.1022)

tau (square root of estimated tau^2 value): 0.3404

I^2 (residual heterogeneity / unaccounted variability): 66.05%

H^2 (unaccounted variability / sampling variability): 2.95

R^2 (amount of heterogeneity accounted for): 0.00%

Test for Residual Heterogeneity:

QE (df = 7) = 21.0896, p-val = 0.0036

Test of Moderators (coefficient 2):

QM (df = 1) = 0.5189, p-val = 0.4713

Model Results:

Estimate SE zval pval ci.lb ci.ub

Intercept 0.9715 0.6905 1.4070 0.1594 -0.3819 2.3249

**Result of Moderator Analysis for Mean Age (PMS condition only)**

Mixed-Effects Model (k = 7; tau^2 estimator: REML)

tau^2 (estimated amount of residual heterogeneity): 0 (SE = 0.0296)

tau (square root of estimated tau^2 value): 0

I^2 (residual heterogeneity / unaccounted variability): 0.00%

H^2 (unaccounted variability / sampling variability): 1.00

R^2 (amount of heterogeneity accounted for): 0.00%

Test for Residual Heterogeneity:

QE(df = 5) = 2.9775, p-val = 0.7035

Test of Moderators (coefficient 2):

QM(df = 1) = 1.9461, p-val = 0.1630

Model Results:

Estimate SE zval pval ci.lb ci.ub

Intercept 0.7069 0.2977 2.3743 0.0176 0.1234 1.2905

**Result of Moderator Analysis for Mean Age (PMDD condition only)**

Inadequate number of studies to conduct this analysis.
